# Game-based learning in orthodontic education: a systematic review

**DOI:** 10.1038/s41405-024-00218-3

**Published:** 2024-07-04

**Authors:** Kawin Sipiyaruk, Patricia A. Reynolds, Theerasak Nakornnoi, Peerapong Santiwong, Rochaya Chintavalakorn

**Affiliations:** 1 Department of Orthodontics, Faculty of Dentistry, Mahidol University, Bangkok, Thailand; 2 Faculty of Dentistry, Oral & Craniofacial Sciences, King’s College London, London, UK

**Keywords:** Dental education, Orthodontics

## Abstract

**Objective:**

To evaluate educational impact of game-based learning (GBL) in orthodontic education.

**Methods:**

A systematic search was undertaken across four databases (Scopus, PubMed, ProQuest Dissertations & Theses Global, and Google Scholar) to identify relevant articles published from January 2000 to December 2023. Additionally, the reference lists of identified literature were examined to further search for relevant literature. The last search was performed on 28 January 2024.

**Results:**

Following the article selection process, seven articles were included in this systematic review, comprising four randomized control trials and three questionnaire surveys. Six articles were assessed to have a moderate risk of biases, whereas one research exhibited a low risk of bias. GBL interventions assessed in five articles were designed in digital format, while one study implemented evaluated traditional learning, and another employed a card game format. Two RCTs indicated a greater effectiveness of GBL in enhancing learner performance compared to traditional learning methods, while one article found no significant difference. Across all articles, positive perceptions of GBL were consistently highlighted at both undergraduate and postgraduate levels.

**Conclusion:**

This systematic review supports the potential of GBL in orthodontic education. The implementation of GBL is recommended to integrate entertaining and educational elements, fostering learner performance within engaging learning environments. However, it is imperative to acknowledge that the overall quality of evidence is limited, primarily due to the moderate risk of biases identified in six of the included articles. Consequently, further high-quality experimental studies are required to validate the effectiveness of GBL in orthodontic education.

## Introduction

Orthodontics is a dental specialty that emphasizes diagnosing, preventing, and managing craniofacial problems and malocclusions, with a variety of fixed and removable orthodontic appliances [1]. World Federation of Orthodontists (WFO) has set the goals for orthodontic education as to develop learner competencies covering both specific and general skills such as lifelong learning and communication skills [2]. Not only a cognitive domain, but also psychomotor skills are required for orthodontists, which include bracket placement and wire bending [3]. These competencies will allow orthodontists to manage orthodontic problems with patient safety and maintain their skills and knowledge.

A variety of teaching and learning approaches should be required for orthodontic education. Lectures, seminars, and clinical practice are generally required for orthodontic training [2]. Case-based learning can also be considered as effective for orthodontic courses [4]. In addition, technology-enhanced simulations including virtual realities appear to be effective learning strategies in orthodontic education, as they allow learners to improve their competence in safe learning environments [5]. Virtual setups could also be used for learning orthodontic treatment planning through tooth movement simulation [6]. A combination of teaching strategies will support learners to achieve expected learning outcomes in orthodontic education.

Game-based learning (GBL) can be considered as another interactive teaching and learning strategy. There appears to be an increasing use of GBL in dental education [7]. This approach allows learners to gain knowledge and skills through feedback provided during task completion [8, 9]. An entertaining component could engage students with their lessons [10]. There is evidence demonstrating effectiveness of GBL in dental education although further high-quality research is not yet sufficient [7, 11, 12]. Considering these arguments, GBL should be recommended for orthodontic training programs.

Albeit the advantages of the GBL, its educational impact over traditional methods has not yet been clearly evident. In addition, although there had been an increase in research evaluating GBL in orthodontic education, its effectiveness has not been yet comprehensively reviewed. Therefore, this systematic review was conducted to evaluate educational impact of GBL in orthodontic education.

## Materials and methods

### Review design

A systematic review was selected to evaluate whether or not GBL can have educational impact in orthodontic training. This method employed scientific procedures to prevent potential biases in systematically reviewing available evidence, enabling researchers to critically synthesize relevant information into comprehensive evidence [13, 14].

### Search strategy

The systematic search was conducted across four electronic databases, which were Scopus, PubMed, ProQuest Dissertations & Theses Global (PQDT), and Google Scholar. In addition, the reference lists of included articles were screened for additional relevant evidence. Gray literature was also sought to encompass research in GBL wherever feasible. Systematic searches were iteratively performed to develop a robust search strategy [15]. The search terms were developed following the PICO approach (Table 1). However, terms for comparison were omitted from the search strategy to enhance sensitivity.

**Table 1:** Search strategy used for the systematic search.

P - Population	orthodontic OR orthodontics OR orthognathic OR “tooth movement” OR “tooth alignment”
I - Intervention	game OR gamification OR gaming OR edutainment OR simulation OR “virtual reality” OR “augmented reality”
C - Comparison	lecture OR traditional learning OR traditional teaching OR passive learning
O - Outcomes	knowledge OR skill OR competence OR competency OR performance OR engagement OR motivation OR satisfaction

### Inclusion and exclusion criteria

All types of empirical studies evaluating GBL in orthodontic education published from January 2000 to December 2023 were considered as inclusion criteria for this systematic review. However, articles were excluded if they focused on GBL designed for patients or laypeople rather than orthodontic learners. Articles without describing details of GBL or without presenting its education impact were excluded. They were also excluded if they were not available in full-text. These inclusion and exclusion criteria are presented in Table 2.

**Table 2:** Inclusion and exclusion criteria for article selection.

Inclusion criteria	Exclusion criteria
• Any types of empirical studies	• Articles relevant to GBL designed for non-orthodontic practitioners, such as surgeons or laypeople.
• Articles evaluating GBL in orthodontic education	• Articles without describing details of GBL Articles without presenting education impact of GBL
• Articles published between January 2000 and December 2023	• Articles not available in full text

### Article selection

Following the systematic search, titles, abstracts, and full-text were independently screened by two researchers (K.S. and R.C.) by considering inclusion and exclusion criteria. Any disagreements on the article selection between the researchers were resolved by discussing and consulting with the third researcher (T.N.).

### Risk of bias assessment for included articles

The risk of bias assessment of included articles can be considered as essential, as the quality of evidence could reflect the strength of systematic reviews. A risk of bias assessment of articles included in this systematic review was performed by a researcher (KS), which was then verified by another one (RC).

The bias assessment of articles with randomized control trials (RCTs) was performed using a revised tool for assessing risk of bias in randomized trials (RoB 2), which included five domains: (1) Randomisation process, (2) Deviations from intended interventions, (3) Outcome data missing, (4) of Outcome measurement, and (5) Selection of reported results [16]. The judgements included “low risk of bias,” “some concerns”, or “high risk of bias” [16]. However, in this systematic review, ‘some concerns’ were labeled as ‘moderate risk of bias’ to facilitate comparison with non-RCTs.

For included articles of non-RCTs, the bias assessment was conducted using the Cochrane Collaboration tool which is ‘Risk of Bias In Non-randomized Studies of Interventions’ (ROBINS-I), which included seven domains: (1) Confounding, (2) Participant selection, (3) Intervention classification, (4) Deviations from intended interventions, (5) Data missing, (6) Outcome measurement, and (7) Selection of reported results, in which the risk of bias was evaluated whether it is low, moderate, serious, or critical [17].

### Data extraction and synthesis

The data retrieved from included articles were extracted in the following aspects: details of GBL, learning subjects, research objectives, methodology, outcome measurement, key findings, and risk of bias assessment (Table 3). A narrative approach was conducted to synthesize the extracted data.

**Table 3:** Data extraction from included articles.

Authors (Year)	Details of GBL	Research objectives	Research methodology	Participants	Outcome measurements	Key findings	Risk of bias
Dhaliwal et al. [18]	Problem-based learning enhanced with audience response system	To determine the impact of ARS in an undergraduate orthodontic course	Cross-over RCT	Dental undergraduates	- User experience - Knowledge retention - Student involvement in class	- Participants had more positive perceptions with the use of ARS. - Knowledge retention was found better with ARS use. - Involvement in class was not affected by ARS.	Moderate
Sakowitz et al. [19]	A simulated patient implemented with virtual reality technology	To compare educational impact in diagnosing and treatment planning of orthognathic cases between VR and 2D prediction tracing method	RCT Participants allocated into two groups: 1. VR 2. 2D tracing	Dental undergraduates	Knowledge and understanding in diagnosis and treatment planning of orthognathic cases	After completing the learning tasks, significant improvements in the test scores were found among participants from both groups. However, no significant difference between the two groups was found.	Low
Huang et al. [20]	Virtual reality system for orthodontic bracket bonding	To investigate user experience in the orthodontic training VR application.	Questionnaire survey following the learning task	Dental undergraduates	User experience	Participants had positive perceptions toward the VR, considered as an enjoyable learning task for training orthodontic bracket bonding.	Moderate
Zhang et al. [21]	Uceph: An online game-based learning for cephalometric training, adopting the pattern of shooting games.	To assess whether Uceph can improve tracing accuracy and speed	RCT Participants allocated into three groups: 1. Uceph 2. Teacher guidance 3. Self-training	Orthodontic resident	- Tracing accuracy - Tracing speed - Self-perception	Uceph offered better performance on tracing accuracy and more positive perceptions. No significant difference between the Uceph and teacher guidance group in terms of tracing speed was found.	Moderate
Chen et al. [22]	Virtual reality to stimulate students to learn orthodontic concepts and treatment process	To evaluate impact of VR compared with PowerPoint for traditional case analysis	RCT Participants allocated into two groups: 1. VR 2. PowerPoint	Dental undergraduates	Learning motivation and user experience	Participants reported higher motivation and more positive perceptions for VR than PowerPoint.	Moderate
Rahmani et al. [23]	Educational game: A quiz game format with 2D animation and interactive feedback for teaching orthodontic lateral cephalometric and dental cast analysis.	To evaluate the attitudes of students toward edutainments	Questionnaire survey following the learning task	Dental undergraduates	User experience	Participants had positive perceptions toward the educational multimedia enhanced with edutainments.	Moderate
Tran and Lipp [24]	Dealodontics: A card game for basic orthodontic concepts	To evaluate learner engagement and game-playing experience.	Questionnaire survey following the learning task	Dental undergraduates	User experience	Participants had positive perceptions toward the card game.	Moderate

## Results

### Articles identified from the search

**Fig. 1:**
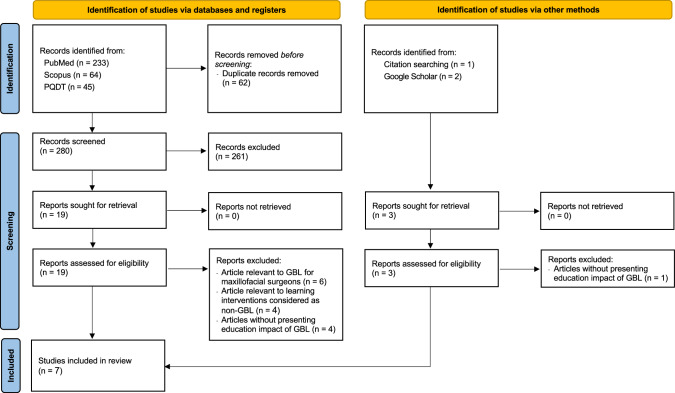
PRISMA 2020 flow diagram of the article selection process.

The flow of article selection process was presented in the PRISMA diagram (Fig. 1), the search through the four electronic databases revealed 342 articles (PubMed = 233, Scopus = 64, and PQDT Global = 45). Following the removal of 62 duplicate records, 280 articles were screened based on the consideration of their titles and abstracts according to the inclusion and exclusion criteria, in which 261 of them were excluded. Together with three articles identified from the citation searching (*n* = 1) and Google Scholar (*n* = 2), 22 full-texts were assessed for their eligibility, where 15 articles were excluded due to being relevant to GBL for maxillofacial surgeons rather than orthodontic practitioners (*n* = 6), being relevant to learning interventions considered as non-GBL (*n* = 4), and no education impact of GBL reported (*n* = 5). Consequently, there were seven full-texts included in this systematic review [18–24].

### Characteristics of articles included in this systematic review

Four articles evaluated GBL using RCTs to compare its educational impact between GBL and traditional learning approaches [18, 19, 21, 22]. Three studies employed a survey design to explore learner perceptions toward the use of GBL without controlled group [20, 23, 24]. Only one article evaluated the educational impact of GBL in orthodontic postgraduates [21], while the remaining research investigated impact in dental undergraduates [18–20, 22–24]. Most of the included studies were assessed to have a moderate risk of biases [18, 20–24], whereas one research article exhibited a low risk of bias [19].

### Characteristics of GBL evaluated in the included articles

GBL in all included articles emphasized on the educational impact on cognitive domain [18–24]. Five articles evaluated GBL in digital format designed specifically for orthodontic education were technology enhanced learning [19–23], one research gamified traditional learning approach with the incorporation of audience response system to traditional [18], and one GBL was a card game format [24]. The topics for GBL were varied, covering basic orthodontic concept [18, 22, 24], model analysis [23], cephalometric tracing and analysis [21, 23], prediction in orthognathic surgery [19], and orthodontic bracket bonding [20].

### Educational outcomes of the interventions in the included articles

#### Performance improvement

Three studies evaluated learner performance following the completion of GBL tasks [18, 19, 21], all of which concluded that GBL could offer cognitive improvement in specific aspects of orthodontic education. However, the expected learning outcomes to be achieved across these GBL interventions varied. One study demonstrated better knowledge retention among dental undergraduates with the implementation of an audience response system as an element of GBL [18]. Another study revealed a higher level of cephalometric tracing accuracy among orthodontic residents following the use of shooting game-based activities, although no significant difference of tracing speed was observed [21]. These two studies indicated that GBL can be effective in enhancing learner performance compared to traditional learning methods in orthodontic education. However, one research found no significant difference in knowledge improvement related to the diagnosis and treatment planning of orthognathic cases [19].

#### User experiences

Six articles investigated user experience toward the use of GBL in orthodontic education contexts, employing questionnaires to assess learner experiences, particularly in terms of enjoyment and engagement, following the learning tasks [18, 20–24]. Among them, three RCTs compared user perceptions between GBL and traditional learning, revealing that participants tended to perceive GBL as more positively in terms of both learning and motivating aspects [18, 21, 22]. However, class involvement of participants was significantly different between GBL and traditional learning [18]. The remaining three articles conducted questionnaire surveys following the GBL activities and found that participants tended to perceive GBL as an effective learning approach, with all studies reporting positive perceptions toward GBL [20, 23, 24]. The findings across the six articles highlighted a notable trend toward favorable perceptions of GBL in orthodontic education at both undergraduate and postgraduate levels.

## Discussion

This systematic review revealed an increasing use of GBL in orthodontic education in recent years, where only one article was published before 2020 [18]. This trend demonstrated a delayed implementation of GBL in orthodontic education, compared to other healthcare disciplines [7]. The rising number of GBL interventions may be attributed to 3D modeling technologies in orthodontic practice [5]. Nearly all GBL interventions evaluated in the included articles were developed for dental undergraduates, while only one study was conducted among orthodontic residents. This could be a result from the fact that orthodontic residents, unlike undergraduate students, are already committed to pursuing their specialty in the field and are highly motivated to acquire knowledge and skills. Postgraduates prioritize the mastery of clinical practice within a specific area as the primary considerations in making decisions about fellowships [25]. Consequently, strategies to promote learning motivation and engagement may be perceived as relatively less important for postgraduates compared undergraduates.

Available evidence has demonstrated that GBL in orthodontic education yields positive educational outcomes, comparable to traditional learning approaches. The necessity of acquiring knowledge and improving skills has become an important component when assessing GBL within healthcare education [7, 26–29]. These outcomes were evaluated through learner performance and self-perceived assessments. Dental students have the opportunity to enhance their knowledge through feedback received following their interactions within GBL environments [30–32]. Technology could enhance GBL to provide immediate feedback enabling interactive asynchronous learning [7]. Certainly, this highlights the positive impact of GBL on orthodontic education by enhancing learner knowledge and competence.

GBL also provides entertaining support, facilitating learner engagement with the learning content. Evidence from all reviewed articles suggests that learners view GBL interventions positively, emphasizing their entertainment value, particularly citing their embedded features such as immediate feedback [18, 23] and competition components [21, 24]. Additionally, a VR format provides an immersive learning environment, reducing distractions from the surrounding environment [19, 20, 22]. These entertainment components motivate learners to actively participate and sustain their interest throughout the learning tasks, facilitating the repetition of a game cycle [33]. Consequently, the integration of entertaining and educational components is essential to engage learners in GBL activities, ultimately leading to the achievement of learning outcomes.

All GBL approaches evaluated in the included articles were primarily designed to enhance cognitive abilities. Despite the hands-on nature of orthodontic practice [2], none of the reviewed articles specifically assessed GBL with a focus on psychomotor skills. Haptic technologies can be integrated into GBL to simulate clinical situations, offering users the opportunity to improve their proficiency in specific tasks [34]. This emphasis on psychomotor skills within GBL could be particularly beneficial for orthodontic residents seeking to gain experience and proficiency in tasks such as wire bending, bracket positioning, or mini-implant placement. Consequently, this expansion of GBL applications to orthodontic postgraduates could enhance skill acquisition and proficiency, with a focus on advanced techniques, supplementing the basic concepts usually addressed at the undergraduate level.

This systematic review was carefully constructed to ensure methodological robustness, where two researchers independently screened and selected identified articles to mitigate potential selection bias. Furthermore, the review had no language restrictions in article selection, thereby minimizing language bias. However, there appears to be heterogeneity in the research outcomes of the included articles, including cognitive improvement, knowledge retention, tracing performance, and learning motivation. Concerning the quality of included articles, only one exhibited a low risk of bias, while the remaining six raised concerns regarding bias. Consequently, additional RCTs with rigorous research design are required to evaluate specific learning outcomes. This is crucial for facilitating meta-analysis aimed at confirming the efficacy of GBL in orthodontic education. Qualitative research should also be performed to investigate in-depth information on how orthodontic residents achieve learning outcomes within the repetition of the game cycle.

## Conclusion

Limited evidence from the articles included in this systematic review demonstrates the potential of GBL in orthodontic education, particularly for cognitive enhancement at the undergraduate level. Students were able to improve their knowledge and comprehension with engagement and motivation through learning activities that integrated entertaining and educational components. However, further research on the application of GBL to enhance skill acquisition and proficiency in orthodontic postgraduates is necessary to ascertain the effectiveness of GBL in orthodontic education.

## Data Availability

The data that support the findings of this study are available from the corresponding author, up-on reasonable request.
